# Hyperacusis Questionnaire as a Tool for Measuring Hypersensitivity to Sound in a Tinnitus Research Population

**DOI:** 10.1155/2015/290425

**Published:** 2015-10-18

**Authors:** Kathryn Fackrell, Constance Fearnley, Derek J. Hoare, Magdalena Sereda

**Affiliations:** ^1^NIHR Nottingham Hearing Biomedical Research Unit, Nottingham NG1 5DU, UK; ^2^Otology and Hearing Group, Division of Clinical Neuroscience, School of Medicine, University of Nottingham, Nottingham NG7 2RD, UK; ^3^School of Medicine, University of Nottingham, Nottingham NG7 2RD, UK

## Abstract

Hypersensitivity to external sounds is often comorbid with tinnitus and may be significant for adherence to certain types of tinnitus management. Therefore, a clear measure of sensitivity to sound is important. The aim of this study was to evaluate the validity and reliability of the Hyperacusis Questionnaire (HQ) for use as a measurement tool using data from a sample of 264 adults who took part in tinnitus research. We evaluated the HQ factor structure, internal consistency, convergent and discriminant validity, and floor and ceiling effects. Internal consistency was high (Cronbach's alpha = 0.88) and moderate correlations were observed between the HQ, uncomfortable loudness levels, and other health questionnaires. Confirmatory factor analysis revealed that the original HQ three-factor solution and a one-factor solution were both a poor fit to the data. Four problematic items were removed and exploratory factor analysis identified a two-factor (attentional and social) solution. The original three-factor structure of the HQ was not confirmed. All fourteen items do not accurately assess hypersensitivity to sound in a tinnitus population. We propose a 10-item (2-factor) version of the HQ, which will need to be confirmed using a new tinnitus and perhaps nontinnitus population.

## 1. Introduction 

Hyperacusis is most commonly defined as increased sensitivity to ordinary environmental sounds that would not usually be bothersome to most individuals [1–3]. Hyperacusis is a broad spectrum condition affecting individuals to various degrees. The main difference between hypersensitivity to sound and conditions such as phonophobia (fear of sound) and misophonia (dislike of sound) is that the latter two usually involve an emotional response to specific sounds [4]. Loudness recruitment (abnormal growth in the perception of loudness) may be a distinct condition or can accompany hyperacusis in people with cochlear hearing loss. Baguley [5] suggested that loudness recruitment can be distinguished from hypersensitivity to sound based on the intensity of the sounds perceived as uncommonly loud (moderate intensity in the case of loudness recruitment and low intensity in the case of hyperacusis). He also notes that loudness recruitment, unlike hyperacusis, does not vary with mood.

Prevalence of hypersensitivity to sound in adults is estimated at 8 or 15% [6, 7]. It can influence emotional well-being, hearing, sleep, and concentration, cause anxiety, and interfere with speech perception in noise [8, 9]. It is estimated that about half of patients with hyperacusis also have a psychiatric or anxiety disorder [10]. Among the possible etiologies of hyperacusis are conditions involving the peripheral auditory system (e.g., Bell's palsy, Ramsay Hunt syndrome, noise-induced hearing loss, Ménière's disease), diseases and syndromes of central nervous system (e.g., headaches, depression, head injury, Williams's syndrome, learning disabilities, spinal problems), and hormonal (e.g., Addison's disease) and infectious diseases (e.g., lyme disease). However, in most cases hypersensitivity to sound has no known cause [3].

Hypersensitivity to sound and tinnitus (perception of sound or noise in the absence of any external acoustic stimulation [11]) are often comorbid. The prevalence of tinnitus among people with hypersensitivity to sound is much higher than in the general population and with estimates of 40% [12], 79% [10], and 86% [13]. Similar to tinnitus, there are several potential pathophysiological mechanisms that might lead to hypersensitivity to sound and similar to tinnitus those mechanisms are not mutually exclusive. Increased prevalence of hypersensitivity to sound in a number of conditions points to 5-hydroxytryptamine (5-HT) dysfunction as one likely mechanism [3, 14]. Interestingly a link between tinnitus and 5-HT dysfunction has also been proposed [3]. One of the postulated functions of 5-HT in the auditory system is modulating central gain [15].

Jastreboff and Hazell [16] described hypersensitivity to sound as a pretinnitus state as it often occurs before the onset of tinnitus. They postulated that hypersensitivity to sound is an effect of an increased gain in the central auditory system. Increased central gain has also been postulated as one of the possible mechanisms of tinnitus generation [17, 18]. Association between hypersensitivity to sound, tinnitus, and peripheral auditory system damage present in stapedectomy, Menieres's disease, and sensorineural hearing loss led to hypotheses assuming peripheral contribution to the generation of hypersensitivity to sound [14].

The strong association between hypersensitivity to sound and tinnitus may have serious implications for research and management of both conditions. Both may have a significant influence on patterns of auditory activity in response to external sounds. The importance of controlling for hypersensitivity to sound in neuroimaging studies of tinnitus has been highlighted in a study by Gu et al. [19] who demonstrated that the increase in neuronal excitability to sounds in tinnitus patients (previously associated with tinnitus) may be ascribed to hypersensitivity to sound rather than to a mechanism specifically related to tinnitus. Several studies point to an association between tinnitus annoyance and the presence of hypersensitivity to sound [20, 21] where tinnitus annoyance is higher in patients with comorbid hypersensitivity to sound. The presence of hypersensitivity to sound can also influence the acceptability and adherence to certain tinnitus management options such as sound therapy. Therefore, a reliable measure of hypersensitivity to sound is important for tinnitus management.

There is no standard protocol for evaluating hypersensitivity to sound. The most common approach includes history taking and measuring uncomfortable loudness levels (ULLs) as a first step in the diagnosis [22]. In people with hypersensitivity to sound ULLs are usually lower than average over all or specific frequencies in both or one ear [5]. According to P. J. Jastreboff and M. M. Jastreboff [22], the average ULLs for patients reporting problems with sound tolerance are between 60 and 85 dB HL, whilst for all other patients ~100 dB HL. ULLs of 70 dB HL or less were suggested by Anari and colleagues as a criterion for diagnosis of hypersensitivity to sound [13, 23]. One common problem with measuring ULLs is high variability and strong dependence of the results on the instruction given. Studies that used different instructions found that the difference in ULLs ranged from 23 to 27 dB HL depending on the frequency [24, 25]. For instance, Dawson Jr. [24] found that, in people with normal hearing and no complaint of hypersensitivity to sound, ULLs were as low as 68 dB HL for 250 Hz, which will be diagnosed as hypersensitivity to sound according to the definition of Anari and colleagues [13]. Test-retest reliability of ULLs has been questioned [26]. Therefore, evidence for the utility of ULLs is mixed.

Patient-reported outcome measures (questionnaires) are used to measure hypersensitivity to sound specific health related quality of life and to diagnose hyperacusis. A small number of questionnaires have been developed to date, including the German Questionnaire on Hypersensitivity to Sound (G Ü F [27]; German version validated in tinnitus patients by Bläsing et al. [28]). The G Ü F assesses the subjective distress associated with hypersensitivity to sound which was considered a better indicator of treatment needs than audiological findings [28]. The German version of the questionnaire has been used in research [29]. Although the English translation of the questionnaire is available, this version has not been validated and has not been used in the clinics or research. The Multiple-Activity Scale for Hyperacusis (MASH) [30] is an interview-based questionnaire which assesses level of annoyance in relation to hypersensitivity to sound. Finally, the Hyperacusis Questionnaire (HQ) [31] was developed and a French version was validated using a general population who did not necessarily complain of sensitivity to sound. During development of the HQ normative data were used to estimate that a score greater than 28 was significantly different to the population total mean score of 15 points and so this was taken to represent “strong auditory hypersensitivity” (hyperacusis) (maximum possible score = 42) [31]. Using exploratory factor analysis, three factors were identified for the HQ (attentional, social, and emotional), but together they only accounted for 48% of the variance; that is, there was a lot of unexplained variance and likely measurement error [32]. Factor loadings were all above 0.3. Minor cross loading in particular in the social factor leads us to question the uniqueness of the subscales. Meeus and colleagues [33] performed validation of a Dutch version of the questionnaire and identified four subscales using exploratory factor analysis. This further calls into question the reliability of the original questionnaire structure identified by Khalfa and colleagues [31]. Exploratory factor analysis is recommended during the development of questionnaires as it explores all possible interrelationships between the set of observed variables without postulating a theoretical structure. However, confirmatory factor analysis is necessary to assess a premediated structure based on theory or/and exploratory factor analysis findings [34, 35]. To date the psychometric properties, in particular the original structure of the HQ, have not been confirmed or assessed in a UK population; no confirmatory factor analysis has been conducted. Yet, the questionnaire is used in tinnitus research studies as a screening tool for exclusion of participants with hyperacusis [36–39] and as an outcome measure of treatment-related change [10, 33], although it was not designed with this purpose in mind [31].

The aim of this study was to empirically evaluate the validity and reliability of the HQ for use as measurement tool in a specific population, that is, adults taking part in tinnitus research. The psychometric validation reported here focuses on evaluating the original three-factor structure of the HQ, particularly item identification with the three factors and the relationship between the three factors and the overall hypersensitivity to sound construct (score), and the reliability (internal consistency), validity (discriminant validity), and responsiveness (floor and ceiling effects) of the HQ using a large UK population of research participants with tinnitus.

## 2. Materials and Methods

### 2.1. Participants and Procedures

The study was a retrospective analysis of data collected during a series of tinnitus research studies conducted at the NIHR Nottingham Hearing Biomedical Research Unit and MRC Institute of Hearing Research between 2008 and 2014. Studies included randomised controlled trials (RCTs) [40, 41], clinical cohort studies [42, 43], an imaging study using magnetoencephalography [38, 39], and a feasibility study [44]. In those studies the HQ was used either as a screening tool for exclusion of participants with hyperacusis [39–41, 43] or for classification of participants [38]. Additional assessments included the Tinnitus Handicap Questionnaire (THQ) [45]; Tinnitus Handicap Inventory (THI) [46]; Beck's Depression Inventory-II (BDI-II) [47]; Beck's Anxiety Inventory (BAI) [48]; Beck's Depression Inventory-Fast Screen (BDI-FS) [49]; uncomfortable loudness levels (ULLs). In some cases participants had completed the eligibility assessments for more than one study; to prevent an overlap in the data, for these participants only one set of questionnaire data was used. In these cases, the most complete dataset was chosen. In total, 264 people with tinnitus (158 male, 106 female) with an average age of 58.7 years (range: 24 to 85 years) completed the HQ and some or most of the assessment questionnaires. Forty people completed ULL assessment.

### 2.2. Missing Data

Only participants who completed the HQ were included (*n* = 264). Due to variability in the eligibility assessments or because of participants being withdrawn at different points in their assessment, not all 264 participants complete all of the other assessments. For the convergent and discriminant validity therefore, we conducted pairwise deletion to enable the use of most data; the effective samples for each comparison are provided in Table 1.

### 2.3. Measures

#### 2.3.1. Hyperacusis Questionnaire (HQ)

The HQ is a two-part questionnaire. The first part consists of binary questions which aim to gather general information of auditory disorders and noise exposure, whilst the second part consists of 14 negatively worded items, which are rated on a 4-point scale: “no” (0 points), “yes, a little” (1 point), “yes, quite a lot” (2 points), and “yes, a lot” (3 points). The total provides the measure of hypersensitivity to sound with higher scores indicating greater sensitivity. The mean global score ranges from 0 to 42 and a global score >28 indicates hyperacusis [31]. Items related to the three subscales can also be summed to provide subscale scores.

#### 2.3.2. Tinnitus Handicap Inventory (THI)

The THI quantifies the impact of tinnitus on daily living [46, 50]. For instance, item 1 asks “Because of your tinnitus is it difficult for you to concentrate?”. Each of 25 items is rated on a 3-point scale: “yes” (4 points), “sometimes” (2 points), and “no” (0 points). The mean global score reflects the sum of all responses with a global score of 100 indicating greatest impact on everyday function. Scores are interpreted using the following categories: slight problem (0–16), mild (18–36), moderate (38–56), severe (58–76), and catastrophic (78–100) [51]. Newman et al. [46] described three subscales measuring functional, emotional, and catastrophic impact of tinnitus. However, the reliability of these subscales has been questioned [52].

#### 2.3.3. Tinnitus Handicap Questionnaire (THQ)

The THQ measures the perceived degree of handicap associated with tinnitus [45]. For example, item 1 asks “I have support from my friends regarding my tinnitus.” For each of 27 items, participants assign a number between 0 (strongly disagree) and 100 (strongly agree) to indicate their agreement. All items are negative descriptors with the exception of two items which are reverse-scored before all the responses are summed and weighted to give a global score out of 100. Kuk et al. [45] identified three subscales ((1) physical, emotional, and social effects; (2) hearing and communication ability; (3) individual's perception of tinnitus) but only subscales 1 and 2 were found to be reliable [45].

#### 2.3.4. Beck's Depression Inventory-II (BDI-II)

The BDI-II measures severity of depressive symptoms [47, 53, 54]. For each of 21 items, participants select one of four statements (scoring 0–3 points) according to how they have felt over the previous two weeks. For example, item 1 measures sadness: (0) “I do not feel sad”; (1) “I feel sad much of the time”; (2) “I am sad all the time”; (3) “I am so sad or unhappy that I cannot stand it.” Higher scores indicate increased levels of depressive symptomatology. Responses are summed to form a global score out of 63, with a score of 31–40 categorised as “severe depression” and a score of over 40 as “extreme depression” [47].

#### 2.3.5. Beck's Depression Inventory-Fast Screen (BDI-FS)

The BDI-FS is a quicker quantitative screen for depression than the BDI, which excludes symptoms possibly related to other medical conditions [49]. Each of the 7 items is rated on a 4-point scale (scoring 0–3 points) with four descriptor statements. Responses are summed to form a global score out of 21, with a higher score indicating a higher level of depression.

#### 2.3.6. Beck's Anxiety Inventory (BAI)

The BAI measures 21 common symptoms of clinical anxiety [48, 55, 56]. Participants indicate the degree to which the particular symptom has bothered them over the previous week by selecting one of four response options (0 to 3). For example, item 1 measures numbness or tingling: (0) “Not at all”; (1) “Mildly; it did not bother me much”; (2) “Moderately; it was very unpleasant, but I could stand it”; (3) “Severely; I could barely stand it.” Again responses are summed to give a global score out of 63. Scores of 0–21 indicate very low anxiety and scores exceeding 36 indicate cause for concern [48, 55].

#### 2.3.7. Uncomfortable Loudness Levels (ULLs)

The ULLs of 40 participants were measured across two of the included studies [38, 39]. ULLs were tested using a 1 kHz pure tone delivered to each ear using an audiometer. Tones were presented for 1 second with 1 second quiet periods and increased in 5 dB steps until the participant responded that the sound had reached an uncomfortable level. All participants had normal hearing thresholds at 1 kHz. ULLs were conducted for both ears and averaged to give a mean ULL value at 1 kHz for each individual.

### 2.4. Statistical Analysis

#### 2.4.1. Factor Structure: Confirmatory Factor Analysis

**Figure 1:**
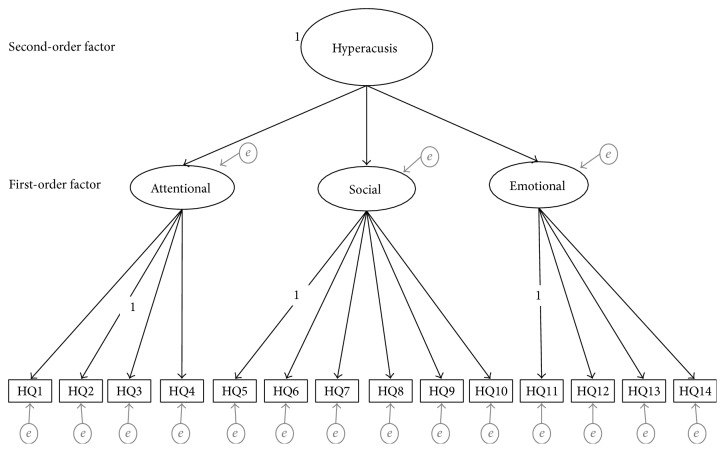
Theoretical 3-factor structure of the Hyperacusis Questionnaire (HQ). The model represents the proposed relationships between the items (observed variables), the first-order factors consistent with attentional, social, and emotional subscales, and the second-order factor consistent with the global measure of “hypersensitivity to sound” (variance fixed at 1). Variance fixed at 1 for second-order factor and items 2, 5, and 11. The unidirectional black arrows represent the direct effects of the second-order factor onto the three first-order factors and the direct effects of the first-order factors onto the observed variables. The fourteen observed variables are represented as HQ1 to HQ14, with all items only associated with their designated factor. The unidirectional grey arrows represent the error variance (*e*) associated with each variable, each freely estimated on their designated factor with zero loadings on the other factors with error variance assumed to be uncorrelated and random (constrained to zero). *e* = residual variance (error and uniqueness terms).

Confirmatory factor analysis (CFA) was conducted on HQ data from 264 patients with tinnitus to test the fit of the 3-factor structure devised by Khalfa et al. (Figure 1) [31]. Following this, to evaluate a modified version of the HQ, the full dataset (*N*: 264) was randomly split into two similar sized independent groups: sample A (50%* N*: 132) and sample B (50%* N*: 132). CFA was conducted on the data from sample A to test the fit of a one-factor structure. Data from sample B were used for exploratory factor analysis (method below). CFA models were specified and estimated using Mplus version 7 [57].

The 3-factor model included (i) one second-order factor consistent with the global measure of “hypersensitivity to sound” (variance fixed at 1) and three first-order factors corresponding to the three HQ subscales (attentional, social, and emotional), (ii) fourteen observed variables, each freely estimated on their designated factor with zero loadings on the other factors with error variance assumed to be uncorrelated and random (constrained to zero).

The one-factor model was specified to include one general factor corresponding to hypersensitivity to sound, with fourteen observed variables corresponding to the 14 items of the HQ and uniqueness variance associated with each item.

All 14 items of the HQ were rated using polytomous scale. The data were modelled accordingly as categorical variables using the robust weighted least squares estimator (WLSMV) in Mplus [57]. Compared to other methods such as maximum likelihood (ML) and weighted least squares (WLS), WLSMV produces robust estimations, in particular robust standard error and adjusted Chi-square test statistics (*χ* ^2^) for categorical data with small sample sizes and nonnormality in the data [58, 59]. In this study since all variables are categorical, WLSMV is estimated using polychoric correlation matrix of the underlying continuous response variables. These latent responses are related to the threshold parameters of the observed categorical variables, in which the thresholds reflect the position on the underlying continuous and normally distributed characteristic that distinguishes the categories of the observed polytomous variable [58]. The factor intercorrelations were initially examined to access the degree in which the factor relates to one another and overlaps in content before the model as a whole was evaluated (the second-order component of the model). Highly correlated factors (>0.85) are evidence of poor distinction between constructs (poor discriminant validity). Weakly correlated factors (<0.30) indicate unrelated content that is potentially measuring an alternative underlying construct [58, 60].

The goodness of fit was determined using WLSMV *χ* ^2^ [61], Comparative Fit Index (CFI) [62], Tucker-Lewis Index (TLI) [63], and weighted root-mean-square residual (WRMR) [57]. An indication of good model fit is a small nonsignificant *χ* ^2^ estimate (*p* < 0.05) that relative to the degrees of freedom is ≤2.0, CFI and TLI estimates that exceed 0.90 (preferably exceeding 0.95) [64], and WRMR values below 0.95. Root Mean Square Error of Approximation (RMSEA) [65] and confidence intervals (CIs) were reported with values of less than 0.05 and CIs within 0.08 indicating acceptable fit [58, 64, 66, 67]. These cut-off points serve as guidelines for acceptable fit for the model that should be evaluated alongside the other CFA findings, that is, the factor loadings [67].

To compare the one and three factor models, the correct *χ* ^2^ difference tests for nested models were assessed using the DIFFTEST command in Mplus [57, 58].

Squared standardised factor loadings provide the basis for interpretation of the factor loading estimates with categorical data. Squared standardised factor loadings (and standardised factor loadings) were therefore examined to evaluate the amount of variance in the underlying continuous response variable explained by the latent constructs (first-order and second-order factors). The strength of these loadings is relative to the amount of variance by the model; therefore, the higher the loading value, the less the error associated with the model and the better the fit. Alternatively, low loadings (<0.4) indicate measurement error and are a potential source of poor model fit [34, 60]. Modification Index (MI) was used to identify any misspecification in the parameters of the model, with values exceeding 10 indicating a source of poor model fit [60]. The Expected Parameter Change (EPC) value was used to identify the magnitude of improvement to model fit if the parameters were freely estimated in a subsequent analysis. Together, these were only used to identify which parameters could be adjusted if they significantly improved model fit and were supported by conceptual foundations [60, 68].

#### 2.4.2. Factor Structure: Exploratory Factor Analysis

Data from sample B were modelled in exploratory factor analysis using the WLSMV estimator and oblique rotations [58, 69]. Following the Kaiser criteria, factors with eigenvalues above 1 were extracted [70]. The scree plot was also examined to confirm factor extraction. Communalities were assessed for each item with communalities below 0.5 taken to indicate a large amount of unexplained variance [32, 58, 71]. Factor loadings were considered meaningful if they exceed 0.40 [34], but to assess cross-loading, the loading estimates should be below 0.30 [72].

### 2.5. Psychometric Properties

The reliability, validity, and responsiveness of the HQ were assessed. All statistical analyses were performed in SPSS (v.22.0).

#### 2.5.1. Reliability: Internal Consistency

Internal consistency was measured as Cronbach's alpha with estimates *α* > 0.7 and *α* < 0.9 taken to indicate acceptable internal consistency [32, 73].

#### 2.5.2. Validity: Convergent and Discriminant Validity

Convergent validity and discriminant validity were evaluated as Spearman's bivariate correlations. Due to the close relationship between tinnitus and hypersensitivity to sound (common problems with concentration/attention, stress, hearing difficulties, participation), the HQ was predicted to moderately correlate with tinnitus questionnaires, that is, moderate discriminant validity. The HQ was predicted to also show moderate correlations with generalised depression and anxiety, because hypersensitivity to sound is associated with both [8, 74].

#### 2.5.3. Responsiveness: Floor and Ceiling Effects

The HQ was not designed for use as an outcome measure; however, some studies still use it for that purpose [33, 40]. Therefore, we looked for floor and/or ceiling effects which would compromise the reliability and responsiveness of the HQ to meaningful changes, although this does not necessarily reflect “real world” change. Response frequency distributions were examined to detect floor or ceiling effects at item level. Floor or ceiling effects were identified as items where more than 15% of respondents rated the lowest or highest possible response option (“no” (0) or “yes, a lot” (3) on a 4-point scale) [32]. Problematic items with floor effects are unlikely to detect reductions in hypersensitivity, whilst items with ceiling effects have limited sensitivity to increases in hypersensitivity.

## 3. Results 

### 3.1. Inspection of the Distribution of Scores

**Figure 2:**
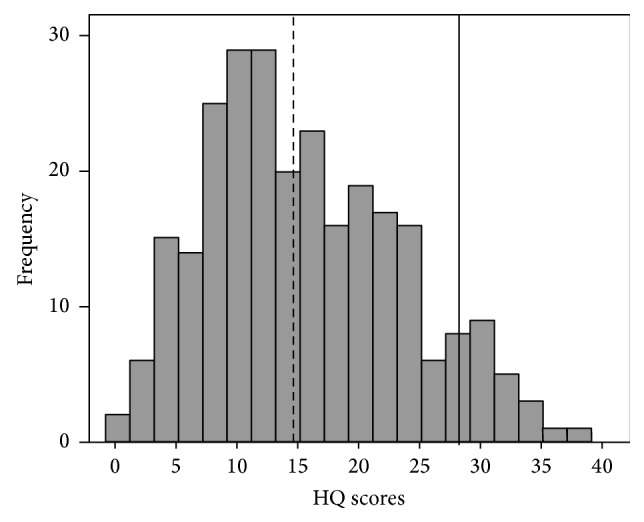
Distribution of Hyperacusis Questionnaire total scores. The diagnostic criterion is represented with a black bold line (—). The mean score for the current study is presented as a black bold dotted line (- - - - -). According to the criteria identified by Khalfa et al. [31], only 7% of participants indicate hypersensitivity, whilst 47% of participants were above our mean score.

Descriptive statistics for all questionnaire measures are shown in Table 1. Mean scores for all the questionnaires were at the lower end of the scoring range, with BDI, BAI, and BDI-FS recording the lowest means relative to their maximum possible score. Scores for tinnitus severity in relation to the THI grading system were moderate (<38/100 in each case). Frequency distributions for global HQ scores are given in Figure 2. The HQ scores were slightly skewed towards the lower end of the scales, with a mean score of 14.7. This mean score was almost identical to the mean questionnaire score (15.0) identified by Khalfa and colleagues [31]. Just under half the participants (124 out of 264) were above 14.7, whilst only 19 out of the 264 participants were above 28 and therefore were identified as experiencing hyperacusis.

### 3.2. Factor Structure: Confirmatory Factor Analysis

#### 3.2.1. Three Factor Structure

First-order factor correlations, standardised factor loadings, standard error, and squared standardised factor loadings for the observed variables and latent constructs are summarised in Table 2. Correlations between the first-order factors (three subscales) were above 0.70 (Table 2), indicating that there was a degree of overlap between the factors.

Model fit was poor; the WLSMV *χ* ^2^ was significant (280.77 (df = 75), *p* < 0.001), and relative to the degrees of freedom, the estimate was significantly higher (3.74) than the critical ratio cutoff (≤2.0). Although the TLI (0.92) and CFI (0.94) were both within the acceptable criteria (marginally below 0.95), both the RMSEA (0.1; CI = 0.09–0.12) and WRMR (1.28) estimates were exceptionally higher than the* a priori* cutoff for establishing adequate fit.

Examination of the squared standardised factor loadings showed that all three first-order factors (attentional, social, and emotional) had high loading values with the second-order factor (over 70% of variance). The loading values for the items ranged from 0.09 to 0.87, with the majority above 0.4. For ten items over 50% of the variance was explained by the first-order factor in which the items are assigned to. For the remaining four items, the standardised factor loadings were low (<0.6), with item 1 below acceptable (0.3). The squared loading values mirrored these loadings (<0.4). The social factor only explained 32% of the variance in items 5 and 6, the emotional factor explained 33% variance in item 11, and the attentional factor only explained an unacceptable 9% variance in item 1. There is a large amount of measurement error that the model cannot explain.

Examination of the modification index revealed the presence of 18 large modification indices (>10). These MIs indicated serious cross-loading between each factor and a number of items. The EPC values indicate that if these parameters were freely estimated, then the improvement to the model would be marginal. Due to the amount of MIs, the small EPC values, and the fact that the model has poor fit statistics, it would make no logical sense to adjust these parameters. A one-factor model might provide a better explanation for the data.

#### 3.2.2. One-Factor Structure

Model fit again was poor; the WLSMV *χ* ^2^ (429.88 (df = 77), *p* < 0.001) and all approximation fit indices failed to meet criteria for a good fit. The squared standardised factor loadings indicated the same problematic items (Table 2). The correct *χ* ^2^ difference tests indicate that the restricted one-factor model significantly degrades the fit of the model (*χ*_diff_^2^(2) = 108.573, *p* < 0.001). These findings suggest that one-factor model does not provide an alternative solution for the data.

#### 3.2.3. Exploratory Factor Analysis

Having removed the four problematic items identified in both CFA models (items 1, 5, 6, and 11), the data from sample B was modelled using WLSMV and oblique rotations estimates. Examination of the eigenvalues (>1) and scree plot revealed a two-factor solution (Table 3). Factor 1 consists of 6 items (attentional items) and factor 2 consists of 3 items (social items). One item (Item 7) did not load onto either factor, with low loading estimates across both (<0.4). Items have loading estimates above the desired criteria and show minimal evidence of cross-loading, except for item 14. For the most part, the communalities were acceptable (>0.50), expect for item 2 and again item 7 which were below < 0.4. Low loading and the low communality suggest that item 7 is unrelated to the underlying construct being measured by the two factors and therefore provides little information on hypersensitivity. Finally the two factors moderately correlated with each other indicating that they are measuring different aspects of hypersensitivity.

### 3.3. Reliability: Internal Consistency

Interitem correlations are presented in Table 4. The correlations ranged from 0.06 to 0.72. Item 1 displayed extremely low correlations with the rest of the items (<0.2), particularly with item 11 (0.06) indicating no relationship between the item contents. The interitem correlations for the social subscale indicate that items 5 and 6 are unrelated (<0.3) to the other items in the subscale indicating poor internal consistency. The rest of the items have low to moderate correlation with each other, suggesting variability in item content. Only one correlation, between item 9 and item 10, indicates high internal consistency (above 0.7). In contrast, Cronbach's alpha estimates for the HQ global score were high (*α* = 0.88) and subscale scores were all above the specified criteria (*α* > 0.7).

### 3.4. Validity

Convergent validity between the HQ and ULLs was moderate (*r* = −0.535) suggesting that the two tools are measuring different constructs with some association. Discriminant validity was as predicted (Table 5). HQ scores showed moderate positive correlations with the two measures of tinnitus severity (THI and THQ) and the three general health measures (BDI, BDI-FS, and BAI). Therefore, with regard to these measures, the HQ demonstrates acceptable discriminant validity indicating that it measures construct(s) that are distinct from tinnitus specific and more general health domains.

### 3.5. Responsiveness: Floor and Ceiling Effects

Response frequency distributions for each HQ item are displayed in Table 6. All fourteen items failed to meet the a priori criterion for acceptable floor and ceiling effects. Twelve items (items 4, 12, 14, 13, 3, 2, 11, 9, 7, 1, 10, and 6) showed floor effects, with 17% to 71% of participants scoring “0.” Item 6 (70%), item 10 (68%), and item 1 (68%) had extreme floor effect with over two-thirds of participants scoring “0.” Two out of the twelve items (items 4 and 12) that showed floor effects also showed mild ceiling effects, with 17% of participants scoring “3.” The remaining two items (items 8 and 5) showed ceiling effects, with 25% and 45% of participants, scoring “3,” respectively. Therefore, these response options are not reliably distinguishing between participants and cannot be considered responsive to changes, at least in this particular population.

## 4. Discussion

The current study evaluated psychometric properties of the HQ in a large UK population of research participants with tinnitus. Despite the high internal consistency estimates, analyses did not confirm the original three factor solution proposed by Khalfa et al. [31] for our UK research population data. Large amounts of cross-loading between the questionnaire items and high correlations between the factors suggested that a one-factor solution is more likely optimal. However, a one-factor solution similarly indicated a poor fit. Four out of 14 items (items 1, 5, 6, and 11) had factor loadings below 0.4 in both models tested potentially suggesting that the wording of these problematic items in relation to this particular population is more likely the cause of poor fit in the three-factor solution. The poor fit could at least in part be due to some population (tinnitus specific) factors. Item 1 asks* “Do you ever use earplugs or earmuffs to reduce your noise perception?”* and although intended to assess attentional component of hyperacusis in a general population [31], within a tinnitus population using ear protection in normal environments is not encouraged, hence the possibility that people with tinnitus will refrain from using earplugs or ear muffs. Item 6 also showed floor effects; it asks* “Has anyone you know ever told you that you tolerate noise or certain kinds of sound badly?”*. The floor effects seen in this item could potentially reflect the management strategy for tinnitus such as sound therapy and exposure to moderate levels of background noise; in particular, tinnitus habituation therapies (e.g., tinnitus retraining therapy) combine education with sound [75, 76]. Item 5 asks “*Do you have difficulty listening to conversations in noisy places?”*. A possible reason it might not fit with the social subscale is that similar difficulties are reported due to hearing loss and tinnitus but not necessarily to hypersensitivity to sound. Finally, item 11 asks* “Do noises or particular sounds bother you more in a quiet place than in a slightly noisy room?”*. Some people with tinnitus will use background noise to “drown out” or mask their tinnitus, while quiet environment can exacerbate their tinnitus and so generally tend to avoid quiet [77]. Consequently, these items were removed and a two-factor solution, with an attentional and social component with the 10 items was identified using exploratory factor analysis.

In terms of convergent and discriminant validity, moderate correlations were observed for all measures suggesting that the HQ is measuring an alternative construct to these measures; in particular, the HQ measures a construct that is different to the sensitivity to loud sounds measured as ULLs. However, due to the differences in measures used to test convergent and discriminant validity (a psychoacoustic test and questionnaires, resp.), it is hard to definitively establish the level of discriminant validity. To provide clarity on this, one recommendation for future studies is to assess convergent validity using a questionnaire measure of hyperacusis.

It is worth noting that only 19 out of 264 participants were classified as hyperacusic according to the criterion of 28 points or more proposed by Khalfa et al. [31]. That indicates the prevalence of hyperacusis in the UK tinnitus research population of about 7.2% which is considerably lower than previously reported for the tinnitus population [10, 12, 13], suggesting that the criterion score might be too high. The criterion score greater than 28 for diagnosing significant hypersensitivity to sound was also questioned by Meeus et al. [33], who reported that most of patients who reported lower tolerance for noise and fear of noise scored below 28 on the HQ.

The HQ was developed to quantify and characterise hypersensitivity to sound and not to be an outcome measure [31]. It is, however, used as an outcome measure [33, 40]. All items showed floor (12 items) or ceiling effects (2 items) with two items showing extreme floor effects where over 60% of participants scored “0.” This indicates that those response options do not reliably distinguish between participants and would not be responsive to changes in severity in this particular population. Therefore, we conclude the 14-item HQ is not a sensitive measure of outcome.

## 5. Conclusions/Recommendations

- The HQ does not provide a valid overall measure of hypersensitivity to sound in the UK population with tinnitus. The structure of the questionnaire was not confirmed. Until an appropriate questionnaire is developed, we recommend the removal of confounding items and evaluation of a 10-item (2-factor) version of the questionnaire in a new tinnitus and perhaps nontinnitus population.
- The diagnostic criterion (28 points) needs to be reevaluated. In order to stratify severity, one suggested method is through the use of anchor questions which can provide external indicators of severity. This strategy has been used in development of the Tinnitus Functional Index and also for identifying meaningful change scores [78–80]. For sensitivity to sound at screening, stratification can be based on response levels in the anchor question, by directly comparing them to the overall score on the questionnaire, providing a practical interpretation of the scores that reflects the patients' opinions.
- A questionnaire measure of sensitivity to sound that is more suitable for use in tinnitus research population should be identified or developed.
- For completeness the HQ needs to be validated in general (including UK) populations, and validation should include test-retest and convergent validity.

## Figures and Tables

**Table 1:** Descriptive statistics for the study measures.

Questionnaire/subscale	Number of items	Total range	Descriptive statistics		Reliability		
			Mean	SD	Range	*α*	*N*
Hyperacusis Questionnaire [31]	14	0–42	14.9	8.0	0–37	0.88	264
Attentional	4	0–12	4.0	2.7	0–10	0.71	
Social	6	0–18	6.1	3.7	0–18	0.75	
Emotional	4	0–12	4.7	3.0	0–12	0.77	
Tinnitus Handicap Inventory (THI) [46]	25	0–100	35.0	21.6	0–94		115
Tinnitus Handicap Questionnaire (THQ) [45]	27	0–100	37.9	17.6	5.6–88.9		195
Beck's Depression Inventory-II (BDI-II) [47]	21	0–63	7.9	7.2	0–30		54
Beck's Depression Inventory-Fast Screen (BDI-FS) [49]	7	0–21	2.0	2.7	0–14		142
Beck's Anxiety Inventory (BAI) [48, 55]	21	0–63	7.0	6.9	0–43		200
Uncomfortable loudness levels at 1 kHz (dB HL)	—	—	87.8	14.3	60–120		40

The maximum score is 42 for the HQ, 100 for THI and THQ, 63 for BDI and BAI, and 21 for the BDI-FS. The reliability alpha (*α*) is presented for the HQ total and subscale scores. *N* = effective samples.

**Table 2:** Standardised factor loadings (standard error), *R*-squared values, and factor correlations for the three-factor model and one-factor model of the Hyperacusis Questionnaire.

	Three-factor model			One-factor model		
	*F*1	*F*2	*F*3	*R* ^2^	*F*1	*R* ^2^
Items						
HQ1	0.30 (0.07)			**0.09**	0.23 (0.10)	0.05
HQ2	0.74 (0.04)			0.54	0.73 (0.05)	0.54
HQ3	0.78 (0.03)			0.60	0.73 (0.05)	0.53
HQ4	0.94 (0.02)			0.87	0.85 (0.03)	0.72
HQ5		0.57 (0.05)		**0.32**	0.55 (0.07)	0.31
HQ6		0.57 (0.06)		**0.32**	0.65 (0.06)	0.42
HQ7		0.75 (0.05)		0.57	0.77 (0.05)	0.59
HQ8		0.73 (0.04)		0.51	0.65 (0.05)	0.43
HQ9		0.83 (0.03)		0.69	0.80 (0.04)	0.64
HQ10		0.84 (0.04)		0.70	0.80 (0.05)	0.64
HQ11			0.50 (0.05)	**0.33**	0.52 (0.07)	0.27
HQ12			0.82 (0.03)	0.73	0.81 (0.04)	0.66
HQ13			0.72 (0.05)	0.60	0.66 (0.06)	0.43
HQ14			0.84 (0.03)	0.66	0.76 (0.04)	0.57
Construct						
Hypersensitivity to sound	0.88 (0.03)	0.86 (0.03)	0.87 (0.03)	—	—	—
*R* ^2^	0.77	0.75	0.75	—	—	—
Factor correlations						
** ** *F*1	1			—	—	—
** ** *F*2	0.75 (0.04)	1		—	—	—
** ** *F*3	0.77 (0.03)	0.75 (0.04)	1	—	—	—

The factor loadings (standard errors) and squared factor loadings (*R*-squared) for the 14 items and the first-order factors (three-factor model only). The values presented in bold have poor associations with their designated factor, all below the recommended cutoff < 0.40. The correlations between the first-order factors were all strong. *R* ^2^ = *R*-squared. *α* = Cronbach's alpha. HQ = Hyperacusis Questionnaire; *F*1 = attentional; *F*2 = social; *F*3 = emotional.

**Table 3:** Exploratory factor analysis: factor loadings, communalities, and eigenvalues for the two-factor extraction.

	Items	*F*1	*F*2	Communality
HQ2	Harder to ignore sounds in everyday situations	**0.48**	0.22	0.38
HQ3	Trouble reading in noise	**0.79**	−0.02	0.61
HQ4	Trouble concentrating in noise	**0.83**	0.11	0.79
HQ7	Particularly sensitive to or bothered by noise	0.37	0.37	0.41
HQ8	Noise unpleasant in certain situations	0.18	**0.62**	0.52
HQ9	Think about the noise before going out	−0.04	**0.97**	0.90
HQ10	Turn down invitation because of noise	0.04	**0.85**	0.75
HQ12	Stress and tired ness reduce ability to concentrate	**0.95**	−0.20	0.75
HQ13	Less able to concentrate at end of day	**0.81**	0.02	0.67
HQ14	Certain sounds cause stress and irritation	**0.49**	0.34	0.51

	Eigenvalues	5.25	1.40	—

The factor loading estimates presented in bold are above the recommended cutoff (>0.4) and indicate which factor the item is associated with. Two items show cross-loading, with estimates above 0.3 on the second factor for item 14, whilst item 7 does not load onto either factor. *F*1 = attentional; *F*2 = social.

**Table 4:** Interitem correlations between all fourteen items of the Hyperacusis Questionnaire.

		Attentional				Social						Emotional			
		Q1	Q2	Q3	Q4	Q5	Q6	Q7	Q8	Q9	Q10	Q11	Q12	Q13	Q14
Attentional	Q1	1													
	Q2	**0.20**	1												
	Q3	**0.12**	0.46	1											
	Q4	**0.17**	0.54	0.69	1										

Social	Q5	**0.16**	0.29	0.31	0.42	1									
	Q6	**0.13**	0.36	**0.25**	0.32	**0.20**	1								
	Q7	**0.15**	0.46	0.37	0.50	**0.21**	0.31	1							
	Q8	**0.16**	0.34	0.32	0.41	0.42	**0.29**	0.36	1						
	Q9	**0.17**	0.38	0.35	0.38	**0.22**	**0.29**	0.35	0.53	1					
	Q10	**0.15**	0.30	**0.24**	0.34	**0.17**	**0.27**	0.32	0.45	0.72	1				

Emotional	Q11	**0.06**	0.32	0.34	0.33	**0.17**	0.30	**0.28**	**0.21**	0.31	**0.23**	1			
	Q12	**0.20**	0.43	0.47	0.55	0.36	**0.29**	0.38	0.35	0.30	0.29	0.38	1		
	Q13	**0.18**	0.34	0.38	0.48	**0.29**	**0.17**	0.31	**0.26**	0.31	0.33	0.36	0.69	1	
	Q14	**0.14**	0.42	0.42	0.53	0.35	0.33	0.54	0.45	0.44	0.38	0.39	0.48	0.42	1

Correlations ranged from extremely low to high. The majority of the items showing low to moderate correlations with each other.

Correlations presented in bold are below the recommended cutoff (0.3), indicating weak relationships between items.

**Table 5:** Correlations between the global scores of the six questionnaire measures.

	HQ	THI	THQ	BDI-II	BDI-FS	BAI
HQ	1					
THI	0.49	1				
THQ	0.40	0.66	1			
BDI-II	0.37	0.45	0.47	1		
BDI-FS	0.32	—	0.21	—	1	
BAI	0.38	0.38	0.28	0.68	0.48	1

The correlations between the HQ and all other measures were moderate indicating acceptable discriminant validity. HQ = Hyperacusis Questionnaire; THI = Tinnitus Handicap Inventory; THQ = Tinnitus Handicap Questionnaire, BDI-II = Beck's Depression Inventory-II, BDI-FS = Beck's Depression Inventory-Fast Screen; BAI = Beck's Anxiety Inventory.

**Table 6:** Response frequency distributions (%) for each Hyperacusis Questionnaire item.

		Frequency of responses for items (%)				Mean	(±SD)
		0	1	2	3		
1	Use earplugs or earmuffs to reduce noise	**67.8**	24.2	4.5	3.4	0.44	(0.74)
2	Harder to ignore sounds in everyday situations	**34.5**	37.9	19.7	8.0	1.01	(0.93)
3	Trouble reading in noise	**31.8**	33.3	22.7	12.1	1.15	(1.01)
4	Trouble concentrating in noise	**17.4**	35.2	30.3	**17.0**	1.47	(0.97)
5	Difficulty listening to conversations in noise	8.7	20.1	28.4	**42.8**	2.05	(0.99)
6	Tolerate noise badly	**70.5**	17.8	5.7	6.1	0.47	(0.85)
7	Particularly sensitive to or bothered by noise	**54.5**	32.2	10.6	2.7	0.61	(0.78)
8	Noise unpleasant in certain situations	13.6	31.8	29.5	**25.0**	1.66	(1.00)
9	Think about the noise before going out	**52.7**	24.2	12.9	10.2	0.81	(1.02)
10	Turn down invitation because of noise	**68.2**	19.7	7.2	4.9	0.49	(0.83)
11	Sounds bother you more in quiet places than noisy	**39.0**	36.4	15.9	8.7	0.94	(0.95)
12	Stress and tired ness reduce ability to concentrate	**19.3**	39.0	25.0	**16.7**	1.39	(0.98)
13	Less able to concentrate at end of day	**29.9**	37.1	22.0	11.0	1.14	(0.97)
14	Certain sounds cause stress and irritation	**22.3**	41.3	23.9	12.5	1.27	(0.95)

Response frequency distributions presented in bold indicate that more than 15% of respondents rated the lowest or highest possible response option. All fourteen items showed either floor or/and ceiling effects (>15%).
